# Hypertriglyceridemia

**DOI:** 10.3390/nu5030981

**Published:** 2013-03-22

**Authors:** Amanda Brahm, Robert A. Hegele

**Affiliations:** Department of Medicine, Schulich School of Medicine and Dentistry, Western University, London, ON N6A 5K8, Canada; E-Mail: aberberi@uwo.ca

**Keywords:** dyslipidemia, polygenic, complex trait, mutation, single nucleotide polymorphism

## Abstract

Hypertriglyceridemia (HTG) is commonly encountered in lipid and cardiology clinics. Severe HTG warrants treatment because of the associated increased risk of acute pancreatitis. However, the need to treat, and the correct treatment approach for patients with mild to moderate HTG are issues for ongoing evaluation. In the past, it was felt that triglyceride does not directly contribute to development of atherosclerotic plaques. However, this view is evolving, especially for triglyceride-related fractions and variables measured in the non-fasting state. Our understanding of the etiology, genetics and classification of HTG states is also evolving. Previously, HTG was considered to be a dominant disorder associated with variation within a single gene. The old nomenclature includes the term “familial” in the names of several hyperlipoproteinemia (HLP) phenotypes that included HTG as part of their profile, including combined hyperlipidemia (HLP type 2B), dysbetalipoproteinemia (HLP type 3), simple HTG (HLP type 4) and mixed hyperlipidemia (HLP type 5). This old thinking has given way to the idea that genetic susceptibility to HTG results from cumulative effects of multiple genetic variants acting in concert. HTG most is often a “polygenic” or “multigenic” trait. However, a few rare autosomal recessive forms of severe HTG have been defined. Treatment depends on the overall clinical context, including severity of HTG, concomitant presence of other lipid disturbances, and the patient's global risk of cardiovascular disease. Therapeutic strategies include dietary counselling, lifestyle management, control of secondary factors, use of omega-3 preparations and selective use of pharmaceutical agents.

## 1. Introduction

Hypertriglyceridemia (HTG) is a common clinical diagnosis, sometimes defined when plasma triglyceride (TG) concentration rises above a threshold value, such as the 90th or 95th percentile for age and sex. HTG frequently co-exists with secondary conditions, including poor diet, alcohol use, obesity, metabolic syndrome, and type 2 diabetes [1,2]. HTG is sometimes classified as “primary”, when a familial or inherited basis is suspected, or “secondary”, when one or more secondary factors contribute to the clinical presentation [1]. Genetic factors can influence the severity of the plasma TG elevation in the presence of a secondary factor [2]. Here we focus on so-called primary HTG, both the rare monogenic and common polygenic forms, in addition to clinical features, diagnosis and management. 

## 2. Clinical Diagnosis of HTG

HTG is usually a biochemical diagnosis, based on fasting plasma TG concentration above a certain cut point [1]. For instance, the 95th percentile for plasma TG is ~250–300 mg/dL (~3.0–3.4 mmol/L) for North American adults. Severe HTG is sometimes diagnosed for fasting plasma TG concentration >1000 mg/dL (>11.2 mmol/L) [1,2,3,4,5,6]. Proposed HTG definitions vary (Table 1). For instance the Adult Treatment Panel III guidelines of the National Cholesterol Education Program has suggested four discrete categories: normal fasting TG is <150 mg/dL (<1.7 mmol/L), borderline high TG is 150–199 mg/dL (1.7–2.3 mmol/L), high TG is 200–499 mg/dL (2.3–5.6 mmol/L) and very high TG is >500 mg/dL (>5.6 mmol/L) [7]. The Endocrine Society has proposed another system with five clinical strata: normal TG is <1.7 mmol/L (<150 mg/dL), mild HTG is 1.7–2.3 mmol/L (150–199 mg/dL), moderate HTG is 2.3–11.2 mmol/L (200–999 mg/dL), severe HTG is 11.2–22.4 mmol/L (1000–1999 mg/dL) and very severe HTG is >22.4 mmol/L (>2000 mg/dL) [8]. Other systems have been proposed, but no single scheme has become predominant in the clinic.

**Table 1:** Hypertriglyceridemia: some proposed clinical definitions.

General clinical definition		ATP Guidelines		Endocrine Society	
Category	Serum TG (mg/dL)	Category	Serum TG (mg/dL)	Category	Serum TG (mg/dL)
Normal	<250	Normal	<150	Normal	<150
Hypertriglyceridemia	250–999	Borderline High	150–199	Mild	150–199
				Moderate	200–999
Severe hypertriglyceridemia	>1000	High	200–499	Severe	1000–1999
		Very high	>500	Very severe	>2000

Abbreviations: TG, triglyceride; ATP, Adult Treatment Panel III of the National Cholesterol Education Program.

## 3. Classification Schemes for Various HTG States

Other classifications for HTG phenotypes are based on qualitative and quantitative biochemical differences in plasma lipoproteins. For instance, a well-established classification system—known as the Fredrickson or World Health Organization (WHO) International Classification of Diseases (ICD) hyperlipoproteinemia (HLP) phenotypes—is based on patterns of lipoprotein fractions. Interestingly, HTG is defining component of five of the six WHO ICD phenotypes; these five are summarized in Table 2 [2,3,9]. The only WHO ICD phenotype that does not have elevated TG levels as part of the definition is familial hypercholesterolemia (FH or HLP type 2A), which is characterized by elevated LDL cholesterol and normal TG levels. FH most often results from mutations in the *LDLR* gene, which encodes the LDL receptor [2,9]. The remaining five HTG-associated HLP phenotypes include the monogenic phenotype called familial chylomicronemia (HLP type 1), and four polygenic “familial” phenotypes, called combined hyperlipidemia (HLP type 2B), dysbetalipoproteinemia (HLP type 3), simple HTG (HLP type 4) and mixed hyperlipidemia (HLP type 5).

**Table 2:** Classification of hypertriglyceridemia (modified Fredrickson).

Name	Primary Lipoprotein Abnormality	Lipid Profile	Clinical manifestations	Population Prevalence
Familial chylomicronemia (HLP type 1)	Elevated chylomicrons	↑↑↑TG ↑TC	- Cutaneous eruptive xanthomata, lipemia retinalis, failure to thrive, recurrent epigastric pain, hepatosplenomegaly, pancreatitis, focal neurologic symptoms	1 in 1 million
Combined hyperlipidemia (HLP type 2B)	Elevated VLDL, Elevated LDL	↑↑TG ↑↑TC	- Physical stigmata such as xanthomas or xanthelasmas are uncommon;	1 in 40
Dysbetalipoproteinemia (HLP type 3)	Elevated IDL, Elevated chylomicron remnants	↑↑TG ↑↑TC	- Tuberous and palmar xanthomata - Elevations in atherogenic IDL results in increased risk for CVD	1 in 10,000
Primary simple hypertriglyceridemia (HLP type 4)	Elevated VLDL	↑↑TG ↑TC	Associated with increased risk of CVD, obesity, DM2, hypertension, hyperuricemia, insulin resistance	1 in 20
Primary mixed hyperlipidemia (HLP type 5)	Elevated chylomicrons, Elevated VLDL	↑↑↑TG ↑↑↑TC	- Similar clinical manifestations as Type I but develops in adulthood - Frequently exacerbated by secondary factors	1 in 600

Abbreviations: as in Table 1, plus: HLP, hyperlipoproteinemia; TC, total cholesterol; VLDL, very-low-density lipoprotein; LDL, low-density lipoprotein; IDL, intermediate density lipoprotein; TG, triglyceride.

These HLP types are defined by the specific classes of TG-rich lipoprotein particles that accumulate in plasma, including chylomicrons, very-low density lipoprotein (VLDL), or intermediate-density lipoprotein (IDL) [2,9]. Simple HTG, namely HLP type 4 is characterized by elevated VLDL concentrations in isolation. But the other HLP types have more complex lipoprotein disturbances. For instance, HLP type 5 is characterized by elevations in both chylomicron and VLDL concentrations. HLP type 3 is characterized by elevated IDL concentrations. HLP type 2B is characterized by elevated VLDL and LDL concentrations. Furthermore, decreased high-density lipoprotein (HDL) cholesterol is commonly seen among patients with all types of HTG. While it was originally thought that the qualitative differences between these HTG-associated phenotypes were genetically based [2,9], recent data indicate that these apparently disparate polygenic phenotypes are similar at the molecular genetic level [3,4,5,6]. 

## 4. Caution Required with the Term “Familial” in HTG States

Before the human genome era, primary HTG was thought to be monogenic, by analogy with other monogenic lipid disorders, namely FH. But while FH results from mutations of strong effect in genes that perturb low-density lipoprotein (LDL) receptor function and visibly segregate with high LDL cholesterol concentrations in family pedigrees, most cases of “familial” HTG are polygenic rather than monogenic disorders [2,3,4,5,6]. This critical distinction is necessary for any current review of this topic.

While cases of HTG cluster in families, HTG within a family does not typically follow classical Mendelian patterns of inheritance. HTG does not consistently show vertical transmission in family pedigrees. But the idea that most HTG states are monogenic has persisted in the literature and textbooks over decades, likely because the term “familial” is included in the names of several classical primary HTG disorders. However, a “familial” disorder should not be confused with a “monogenic” disorder: while many HTG cases are familial, they are usually not monogenic [2,3,4,5,6]. 

## 5. Monogenic HTG: Familial Chylomicronemia (HLP Type 1)

One HTG phenotype is truly monogenic, namely familial chylomicronemia, also known as HLP type 1, which is characterized by the pathological persistence of chylomicrons after a fasting period of 12–14 h [1,2,3]. The remainder of the lipoprotein fractions are normal or low in HLP type 1. The estimated population prevalence of this rare phenotype is ~1 in 1 million [1,2,3]. 

### 5.1. Clinical Features of Chylomicronemia

Development of physical findings in HTG is less common today that in the past, likely due to earlier diagnosis and treatment. Presence of physical findings is generally related to the degree of TG elevation. Very high TG levels (>1000 mg/dL) are associated with chylomicronemia, since these particles have the highest capacity for carrying TG in their core. TG levels >1000 mg/dL (>11.2 mmol/L) are almost always due to elevated chylomicrons. Familial chylomicronemia often presents during infancy or childhood, and generally becomes manifest by adolescence [1,10,11]. Clinical features include failure to thrive, eruptive xanthomas over extensor surfaces and buttocks, lipemia retinalis, hepatosplenomegaly, recurrent abdominal pain, nausea and vomiting, and risk of acute pancreatitis [10,11]. Less common clinical features include intestinal bleeding, pallor, anemia, irritability, diarrhea, seizures and encephalopathy [10,11]. 

Eruptive xanthomas appear on extensor surfaces of the extremities, the buttocks and the shoulders as raised crops of small yellowish papules encircled by erythematous halos [12]. Xanthomas erupt when plasma TG is severely elevated, and gradually disappear over weeks to months as TG levels improve [13]. Microscopically, xanthomas contain lipid-laden macrophages (foam cells) within the superficial reticular dermis, as well as infiltrations of lymphocytes and neutrophils [12].

Lipemia retinalis refers to a whitish-pink appearance of retinal vessels on fundoscopic examination, and is due to the presence of chylomicron-rich serum. This finding is more likely to be present when TG > 3000 mg/dL (>33.6 mmol/L) [1]. Vision is unaffected [13]. 

Hepatosplenomegaly is also related to the degree of TG elevation and results from lipid accumulation within cells of the reticuloendothelial system. Hepatosplenomegaly is rapidly reversible with correction of plasma TG levels [1]. 

Patients with familial chylomicronemia have increased lifelong risk of recurrent pancreatitis [14]. This risk increases when TG >1000 mg/dL (>11.2 mmol/L) and is greatest with TG levels >2000 mg/dL (>22.4 mmol/L) [15]. Interestingly, some patients remain asymptomatic when TG levels are much higher than 2000 mg/dL. Pancreatitis due to HTG can be serious and sometimes fatal. Beyond the acute abdominal discomfort, complications include development of chronic pancreatitis, pancreatic insufficiency, pancreatic necrosis, pancreatic abscess or pancreatic pseudocyst [16]. 

It has been suggested that the pathophysiology of HTG-induced pancreatitis is related to increased activity of exocrine pancreatic lipase, which ectopically mediates hydrolysis of circulating TG within the pancreatic vasculature [1,16]. The partially hydrolyzed fatty acids are thought to be injurious to acinar cells, leading to activation of trypsinogen and local autodigestion of pancreatic tissue [1,16]. Increased chylomicrons might further worsen the process by causing capillary plugging and local ischemia [1,16]. 

Cardiovascular disease (CVD) risk is inconsistently associated with familial chylomicronemia. Younger patients with chylomicronemia are less prone to develop CVD than patients with other lipid disorders [17]. Autopsies of some HLP type 1 patients showed no significant burden of atherosclerosis [18], possibly because chylomicrons are too large to penetrate the endothelial surface [18]. In addition, LDL cholesterol is relatively low in patients with chylomicronemia [18]. Small case studies suggest that some patients with chylomicronemia can still develop premature atherosclerosis [18]. However, the presence of atherosclerosis in this situation could have been due to pro-atherogenic effects of modified chylomicron remnants, or to the impact of low HDL cholesterol in these patients [18]. Patients with premature atherosclerosis are the exception rather than the rule in chylomicronemia [17]. 

### 5.2. Laboratory Features of HLP Type 1

Plasma from individuals with HLP type 1 appears lipemic: turbid and milky [10]. If allowed to settle overnight, it develops a cream-like supernatant above a virtually clear infranatant [1]. Fasting serum TG concentration is generally >1000 mg/dL (>11.2 mmol/L), and sometimes can exceed 10,000 mg/dL (112 mmol/L) [18]. Concomitant lipid abnormalities include a modest elevation in serum total cholesterol, with decreases in LDL and HDL cholesterol [1]. 

### 5.3. Molecular Basis of HLP Type 1

Mutations in at least five different genes cause familial chylomicronemia (Table 3): namely *LPL*, *APOC2*, *APOA5*, *GPIHBP1*, and *LMF1* genes. These mutations are inherited in an autosomal recessive fashion and lead to chylomicronemia [3]. Of these, lipoprotein lipase (LPL) deficiency due to mutations in the *LPL* gene is the most common, with an estimated prevalence of 1 in 10^6^, but has a carrier frequency of 1 in 40 persons in some founder populations [2]. Before DNA testing was available, LPL deficiency was diagnosed by the absence of LPL activity in plasma collected after intravenous injection of heparin [17,18,19]. The diagnosis can now be made by DNA sequence analysis, which shows the presence of mutations on both *LPL* alleles [20]. More than 100 *LPL* mutations present in the simple homozygous or compound heterozygous state have been reported to cause LPL deficiency [20].

**Table 3:** Genes associated with autosomal recessive familial chylomicronemia.

Gene	Disease prevalence	Age of onset	Molecular basis
Lipoprotein lipase (*LPL*)	1 in 1 million (95% of cases)	Infancy or childhood	Severely reduced or absent LPL enzyme activity
Apolipoprotein C-II (*APOC2*)	<20 families described	Adolescence to adulthood	Absent or non-functional apo C-II
Glycosyl-phosphatidyl-inositol-anchored HDL-binding protein (*GPIHBP1*)	<5 families described	Later adulthood	Absent or deficiency in GPIHBP1
Apo A-V (*APOA5*)	<5 families described	Later adulthood	Absent or defective apo A-V
Lipase maturation factor-1 (*LMF1*)	<5 families described	Later adulthood	Defective or absent LMF1

Abbreviations: as in Table 1, Table 2.

Interestingly, the 4 genes that are associated with monogenic HTG besides *LPL* encode products that affect activity, assembly or transport of LPL. For instance, apo C-II is an essential LPL co-activator absolutely required for TG-rich lipoprotein hydrolysis, thus homozygous mutations in *APOC2* cause apo C-II deficiency [21]. Apo A-V is required for efficient lipolysis of TG-rich particles by LPL [22], although its mechanism of action is still not fully understood. Homozygous mutations in *APOA5* causing apo A-V deficiency cause severe HTG [22]. Homozygous mutations in genes that are required for efficient assembly and transport of LPL, including *GPIHBP1* [23] and *LMF1* [24], also cause monogenic HTG. Both *LPL* and *APOA5* genes also harbor common polymorphisms (see Table 4) that are associated with plasma TG levels and HTG [25,26,27,28].

However, many patients with chylomicronemia—perhaps 30%—have no mutation in any of these genes [3]. This suggests that other genes can predispose to HLP type 1. The basis of disease in such patients may soon be resolved using next generation sequencing methods or other new genomic technologies.

**Table 4:** Common DNA polymorphisms associated with hypertriglyceridemia.

CHR	Gene	SNP	Risk allele	OR (95% CI)	*P*-value
11	*APOA5*	rs964184	G	3.43 (2.72–4.31)	1.12 × 10^−25^
2	*GCKR*	rs1260326	T	1.64 (1.36–1.97)	1.97 × 10^−7^
8	*LPL*	rs12678919	A	2.21 (1.52–3.22)	3.5 × 10^−5^
8	*TRIB1*	rs2954029	A	1.50 (1.24–1.81)	3.8 × 10^−5^
1	*ANGPTL3*	rs2131925	T	1.51 (1.23–1.85)	1.0 × 10^−4^
7	*MLXIPL*	rs7811265	A	1.63 (1.25–2.13)	3.3 × 10^−4^
4	*KLHL8*	rs442177	T	1.36 (1.13–1.64)	1.5 × 10^−3^
10	*CYP26A1*	rs2068888	G	1.29 (1.08–1.55)	5.9 × 10^−3^
19	*CILP2*	rs10401969	T	1.72 (1.16–2.54)	6.8 × 10^−3^
2	*APOB*	rs1042034	T	1.28 (1.02–1.61)	0.032

Abbreviations: CHR, chromosome; SNP, single nucleotide polymorphism; OR, odds ratio for hypertriglyceridemia per risk allele; CI, confidence interval; *APOA5*, gene encoding apolipoprotein A-V; *LPL*, gene encoding lipoprotein lipase; *TRIB1*, gene encoding Tribbles homolog 1; *ANGPTL3*, gene encoding angiopoietin-like protein 3; *MLXIPL*, gene encoding MLX interacting protein-like 1; *KLHL8*, gene encoding Kelch like protein 8; *CYP26A1*, gene encoding cytochrome P450 26A1; *CILP1*, gene encoding cartilage intermediate layer protein 2; *APOB*, gene encoding apolipoprotein B [25,26,27,28].

### 5.4. Treatment of HLP Type 1

Treatment of patients with familial chylomicronemia follows the general principles for management of HTG. This includes dietary and lifestyle interventions, control of secondary factors, and pharmacological therapies (see Table 5, Table 6). Unfortunately current drug therapies that are effective for milder HTG states are relatively ineffective for familial chylomicronemia [1]. Also, because of the severely elevated TG and imminent risk of pancreatitis, further specific treatment is indicated for HLP type 1 patients, starting with dramatic restriction of fat intake. 

**Table 5:** Therapeutic goals and treatment strategies in hypertriglyceridemia.

Triglyceride Level	Therapeutic Goal	Therapeutic Strategies
Borderline high (150–199 mg/dL)	Primary: Achieve LDL-C target	- Focus on non-pharmacologic strategies - Rule out and treat secondary factors
High (200–499 mg/dL)	Primary: Achieve LDL-C target Secondary: Achieve non-HDL-C goal, which is 30 mg/dL higher than LDL-C goal	- Implement non-pharmacologic strategies - If LDL-C is close to goal, titrate statin dose to achieve both LDL-C and non-HDL-C targets - If LDL-C is at goal, but non-HDL-C is still elevated, titrate statin dose or add: fibrate niacin omega-3 fatty acid
Very high (≥500 mg/dL)	Priority: reduce triglycerides to prevent acute pancreatitis Other goals: achieve LDL-C and non-HDL-C goals once pancreatitis risk decreased	- Consider: fibrate (preferred) niacin omega-3 fatty acids - Institute non-pharmacologic therapies at the same time as pharmacologic therapies

Abbreviations: HDL-C, high-density lipoprotein cholesterol; LDL-C, low-density lipoprotein cholesterol.

**Table 6:** Managing hypertriglyceridemia in special situations.

Situation	Non-pharmacologic management	Medical management options
Type 1 or V HLP	Diet is mainstay of therapy: <10%–15% calories from fat Use medium chain triglycerides Supplement with essential fatty acids (e.g., walnut oil) Supplement fat-soluble vitamins	Fibrates or niacin may be tried, but may not be very effective. LPL-based gene therapies may be helpful (future). Plasma transfusions may transiently help patients with *APOC2* mutations. Gemfibrozil in the 3^rd^ trimester in pregnancy in HLP type 1. Apheresis is of questionable value.
Acute pancreatitis with severe hypertriglyceridemia	- NPO - Supportive measures - Address secondary or exacerbating factors such as alcohol intake or glycemic control - When PO intake possible, use low fat and low simple carbohydrate diet	- When PO intake possible, use pharmacotherapy with fibrate, niacin, or omega-3 fatty acids. - Insulin therapy when appropriate. - Apheresis is of questionable value.

Abbreviations: HLP, hyperlipoproteinemia; LPL, lipoprotein lipase; NPO, nil per os; PO, per os.

Fat intake targets have not been prospectively evaluated in HLP type 1, but clinical experience confirms the effectiveness of fat restriction. Advice ranges from restriction of total fat intake to <50 g per day, or <25% of daily caloric intake, to stricter levels of <20 g per day, or <15% of total daily caloric intake [1,7]. Unfortunately, such restrictions are challenging, and success in adherence and outcomes has been variable. 

Identifying and managing concomitant secondary causes of HTG is important in HLP type 1. Other elements of management include control of weight, reducing or eliminating alcohol intake, and eliminating use of exogenous estrogens and other medications such as corticosteroids and retinoids [1]. Pregnancy, hypothyroidism, diabetes and chronic renal failure can also worsen HTG and increase pancreatitis risk [1]. 

While case reports suggest that plasmapheresis might help reduce TG [29], this treatment is expensive and requires specialized equipment and personnel [29]. Also, in our experience, patients with severe chylomicronemia who are treated in hospital conservatively with cessation of oral intake of calories, and parenteral fluid replacement show just as rapid improvement in plasma TG levels (reduction by half every 48–72 h) as patients treated with plasma exchange or plasmapheresis [1].

Finally, gene therapy has been studied in patients with familial chylomicronemia. Specifically, expression of a recombinant virus containing the human hyper-functional LPL mutant S447X showed promise in animal models [30]. Early clinical trials in human subjects using intramuscular injections of the recombinant LPL induced local LPL expression and were associated with a transient reduction in plasma TG levels [31]. This treatment (trade name Glybera) was recently approved by the European Medicines Agency for the treatment of HLP type 1. 

## 6. Polygenic or Complex HTG

Polygenic HTG has a complex genetic etiology. First, certain common small effect variants (single nucleotide polymorphisms or SNPs) are consistently overrepresented in the genomes of adult patients with all subtypes of HTG [3,4,5,6]. Second, the genetic pool of adult HTG patients is enriched for rare heterozygous large-effect mutations within genes that are associated with elevated plasma TG levels [3,4,5,6]. Finally, secondary factors can push a genetically susceptible individual over the edge metabolically, resulting in clinical presentation [1].

Our research suggests that HLP type 4 is the foundation of all HTG states. HLP type 4 results from the accumulation of both common and rare genetic variants that increase a patient’s susceptibility to develop HTG due to increased levels of VLDL particles [26]. Patients with HLP type 5 have the same genetic predisposition as HLP type 4, but carry an additional burden of DNA variants together with secondary or metabolic stressors [26], causing chylomicrons to accumulate in plasma together with VLDL. Genetically, most patients with HLP type 3 are essentially HLP type 4 patients with the extra contribution of the *APOE* E2/E2 genotype; the E2 allele of apo E binds poorly to cell surface receptors, resulting in accumulation of VLDL remnants [26]. Finally, the presence of common LDL-associated alleles on top of polygenic HTG susceptibility pushes the clinical phenotype in the direction of HLP type 2B, which is characterized by increased levels of both LDL and VLDL particles [26].

The Global Lipids Genetics Consortium identified 32 common genetic variants or SNPs that raise plasma TG levels slightly in healthy people [25]. The largest effects are for SNPs within the *APOA5*, *LPL*, *GCKR* and *APOB* genes, for which the deleterious alleles each raise TG levels by 4–16 mg/dL in the general population [25]. We found that these same SNP alleles increase HTG risk by ~1.5–3-fold in our HTG patients [26,27,28]. The top 10 common SNP alleles that are associated with HTG risk are shown in Table 4. A patient’s total burden of TG risk alleles can be added together to create a “genetic risk score” for HTG susceptibility [26,27,28]. 

HTG patients as a group have significantly higher genetic risk scores than normolipidemic patients [26,27,28]. A very high or very low genetic risk score can discriminate between HTG and normolipidemic subjects at the extremes of the distribution; however there is substantial overlap of scores between patients and healthy subjects in the middle of the distribution [3,4,5,6]. Nonetheless, while the genetic risk score may have limited clinical utility in many cases, its derivation and calculation has helped define the principle that the HTG population has a higher burden of small-effect common SNPs than the normal population [3,4,5,6].

Furthermore, the pool of HTG patients also has a higher burden of rare large effect variants than individuals with normal TG levels: the proportions of patients are ~15% and ~6% for HTG patients and normolipidemic individuals, respectively [26,32]. While the difference in these percentages is significant, testing for these mutations is not diagnostic for the development of HTG in any particular individual, since most HTG patient do not carry one and since some normolipidemic people do carry one. Also, these rare mutations generally do not co-segregate with TG levels in family pedigrees. Together with the clustering of common SNPs in HTG patients, this clustering of rare variants reveals the complexity of a polygenic trait [26,27,28], which is more relevant for understanding pathophysiology than for any potential clinical application. 

## 7. Secondary Factors Contributing to Polygenic HTG

Secondary non-genetic factors associated with HTG are covered in depth elsewhere [1] but briefly, these include: obesity, metabolic syndrome, diet with high positive energy-intake balance and high fat or high glycemic index, alcohol consumption, diabetes (mainly type 2), renal disease (uremia or glomerulonephritis), pregnancy (particularly in the third trimester), paraproteinemia, systemic lupus erythematosis, and some medications, including corticosteroids, oral estrogen, tamoxifen, thiazides, non-cardioselective beta-blockers, bile acid sequestrants, cyclophosphamide, antiretroviral drugs, and second generation antipsychotic agents. 

Of these secondary causes, the “metabolic syndrome” is a special case because increased TG levels are part of the definition of the disorder [7]. Although it has not been specifically tested experimentally, it is possible that the same genetic variants that predispose to HTG in general also predispose to the elevated TG component of the metabolic syndrome. Furthermore, there is no evidence that differences in genetic susceptibility to the metabolic syndrome overall, or to any of its specific components, would predict differential response to interventions, although this is a hypothesis that could be tested in the future.

## 8. Simple Primary Hypertriglyceridemia (HLP Type 4)

In our clinic for some years now, we have been using the term “simple primary HTG” for the disorder formerly known as “familial HTG” or HLP type 4. This relatively common phenotype is characterized by high TG levels due to an isolated elevation of VLDL particles, which results from both overproduction and decreased elimination of these particles [6,7,8]. Susceptibility to simple HTG results from a heterogeneous group of mechanisms that cause elevations in VLDL [3,4,5,6,28]. TG levels between 300 and 900 mg/dL (3.4 and 9.9 mmol/L) due to isolated elevation of VLDL particles are seen ~5% of adults [1]. Depressed HDL cholesterol levels are often associated with HTG [1]. At the higher end of the TG range for this condition, serum may also appear turbid on examination due to the presence of large VLDL particles [1]. 

Simple primary HTG is associated with an increased risk of CVD, obesity, insulin resistance or frank diabetes, and is associated with hypertension and hyperuricemia [1]. With an additional metabolic stress, simple HTG patients can deteriorate into mixed hyperlipidemia (HLP type 5), with fasting chylomicronemia. Generally, the TG levels resulting from VLDL excess in simple primary HTG are not high enough to cause pancreatitis [15]. 

The molecular basis for simple primary HTG follows the polygenic architecture described above, sometimes with the presence of one or more secondary factors that can force expression of the phenotype in a genetically susceptible person [3,4,5,6,31]. Treatment of simple HTG follows the general strategy outlined in Table 5, Table 6.

## 9. Dysbetalipoproteinemia (HLP Type 3)

Dysbetalipoproteinemia, also known as HLP type 3, is characterized by increased serum TG and cholesterol rich lipoprotein remnants—essentially IDL and chylomicron remnants, sometimes collectively called beta-VLDL particles [1,33]. HLP type 3 is mainly caused by homozygosity for binding-defective apo E2 isoform on a background of genetic susceptibility to HTG that resembles HLP type 4 [2,9,33] and is seen in ~1 in 10,000 people [1,33]. The condition generally does not present until adulthood in men and in the post-menopausal years in women [1,33]. 

HLP type 3 patients are now identified and treated early, so few have the classical physical stigmata. Untreated patients above age thirty can present with tuberous or tuberoeruptive xanthomas on the extensor surfaces of extremities (elbow and knees and occasionally the buttocks) [1,33]. Planar or palmar crease xanthomas are also noted [33]; these appear as orange lipid deposits seen in the crease areas of the palm and are pathognomonic of HLP type 3, however they are not present in all individuals with the condition [33]. HLP type 3 patients have increased risk of both coronary artery disease and peripheral vascular disease [1,33]. 

Dysbetalipoproteinemia often requires secondary factors for overt disease expression. These include additional genetic susceptibility variants, or other hormonal or environmental factors, such as the presence of disorders such as obesity, type 2 diabetes or hypothyroidism [33]. 

HLP type 3 patients typically present with elevated total cholesterol levels, generally 250–450 mg/dL (6.5–11.6 mmol/L) with elevated TG in the 250–900 mg/dL (2.8–10 mmol/L) range [1,33]. Levels of total cholesterol and TG are roughly equally elevated [1]. Directly measured LDL cholesterol is low due to disrupted processing of VLDL to LDL [1,2,33]. When fractionation methods, such as ultracentrifugation and electrophoresis are available, the presence of a broad beta band or of IDL are both suggestive of HLP type 3. An elevated VLDL cholesterol to total TG ratio (>0.3) plus apo E2/E2 homozygosity are often pathognomonic for HLP type 3 [1,2,33]. 

Similar to other HTG states, HLP type 3 is a polygenic trait. But some patients can have additional genetic variants or mutations that affect the normal function of apo E [1,33]. For instance, a minority (<5%) of HLP type 3 patients has rare dominant mutations in *APOE* [1,8], which may not require any secondary factors to be present for expression of the HTG phenotype [8]. Such *APOE* mutations impair hepatic uptake of chylomicron remnants and IDL particles, and also impair conversion of VLDL and IDL to LDL particles [1].

Treatment of HLP type 3 follows the general strategy for HTG management (Table 5, Table 6). Some HLP type 3 patients respond very well to weight loss, reduced fat diets and control of secondary factors, such as type 2 diabetes and hypothyroidism [1]. In our experience, these patients also respond well to most pharmacological treatments, including omega-3 fatty acids, fibrates, niacin, and statins.

## 10. Mixed Hyperlipidemia (HLP Type 5)

Mixed hyperlipidemia, or HLP type 5, like HLP type 1, is characterized by presence of chylomicrons after 12–14 h of fasting [1,2]. But in addition, HLP type 5 has elevated levels of VLDL particles, like HLP type 4. The phenotype is essentially a more extreme form of HLP type 4, in which chylomicrons accumulate during fasting. 

HLP type 5 has a population prevalence of ~1 in 600 [1,2]. A key distinguishing feature between mixed hyperlipidemia and familial chylomicronemia is the age of onset of presentation. Patients with familial chylomicronemia typically present in childhood or adolescence, whereas mixed hyperlipidemia patients typically present in adulthood [1]. Inheritance pattern is variable, with the phenotype thought to be triggered in patients with an underlying genetic susceptibility coupled with the influence of environmental and hormonal exposures [1,34,35,36]. 

Signs and symptoms are similar to those seen in familial chylomicronemia, with eruptive xanthomata, lipemia retinalis, hepatosplenomegaly, increased pancreatitis risk [1] and some neurological symptoms, such as the inability to concentrate [34,35,36]. 

The laboratory findings in mixed hyperlipidemia are similar to those seen with familial chylomicronemia, with an elevated fasting serum level of chylomicrons, with TG typically >1000 mg/dL (>11.2 mmol/L), together with elevated levels of VLDL particles [1,8]. Plasma appears turbid, and develops a creamy supernatant when allowed to stand overnight [1,8,9]. Infranatant can also be turbid. Patients with mixed hyperlipidemia also have associated elevations in total cholesterol, and often other lipoproteins, particularly VLDL, which are not present in familial chylomicronemia [1,2]. 

## 11. Combined Hyperlipidemia (HLP Type 2B)

Combined hyperlipidemia, also known as HLP type 2B, is characterized by increased serum TG and LDL cholesterol [1,36,37]. It affects ~1 in 40 people [1,36,37]. The diagnosis is made based on the combined elevations of TG and LDL cholesterol, often together with depressed HDL cholesterol, in the index patient and classically in at least one first degree relative. However, as mentioned above, the inheritance does not follow a discrete Mendelian monogenic pattern. In a small minority of kindreds, the combined hyperlipidemia phenotype appears to segregate as an autosomal dominant trait (often with variable penetrance), but the molecular cause remains unknown. 

This condition was previously referred to as “familial combined hyperlipidemia”, but as mentioned for other HTG phenotypes that had earlier been defined with the term “familial”, this well-known disorder, while familial, is not monogenic in most instances. The reason that it clusters in families, similar to other familial HTGs, is due to the aggregation of many independent small effect polymorphisms within family units. Further, while “familial combined hyperlipidemia” was previously considered to be primarily a disorder characterized by overproduction of apo B containing lipoproteins, the types of genes that harbor polymorphisms appear to be involved in both production and degradation of lipoproteins [2,3,4,5,6]. 

Patients with combined hyperlipidemia have increased risk of atherosclerotic vascular disease [1,36,37]. Combined hyperlipidemia sometimes requires secondary factors for overt disease expression. These include poor diet, obesity, type 2 diabetes or hypothyroidism [37]. 

Patients with combined hyperlipidemia typically have elevated total cholesterol levels, generally 250–450 mg/dL (6.5–11.6 mmol/L) with elevated TG in the 250–900 mg/dL (2.8–10 mmol/L) range [1,36,37]. Directly measured LDL cholesterol is also elevated, typically >90th percentile for age and sex (>160 mg/dL or 4.1 mmol/L) [1,37]. 

Combined hyperlipidemia is a polygenic trait, like most other HTG states. These patients have a background of increased genetic susceptibility similar to that seen in simple HTG (HLP type 4). But in addition, combined hyperlipidemia patients also carry genetic variants or mutations that raise LDL cholesterol [6,9,28]. This complex genetic susceptibility tends to cluster in families, but because the susceptibility alleles are on different chromosomes, the susceptibility is not transmitted in a clear Mendelian manner. The complex pattern of susceptibility alleles also explains why some family members display only certain components of the combined dyslipidemia pattern, namely isolated or some combination of high LDL cholesterol, high TG or depressed HDL cholesterol. The clustering of genetic susceptibility and secondary factors in families means that biochemical screening of family members and counselling are still indicated once an index case has been identified. In our experience, combined hyperlipidemia patients are relatively refractory to usual treatments, and may require higher doses and combinations of lipid lowering agents, focusing on statins, which are the centerpiece of therapy for reduction of the LDL component of the phenotype.

## 12. Therapeutic Strategies for HTG

According to the NCEP ATP III guidelines, lowering LDL cholesterol is the primary management goal for dyslipidemia. However, attaining a non-high-density lipoprotein cholesterol value within 30 mg/dL of the LDL cholesterol target is advised as a secondary treatment target in patients with TG levels ≥200 mg/dL [7]. Non-HDL cholesterol represents the atherogenic component of the lipid profile, consisting of the sum of LDL and VLDL, and is calculated as total cholesterol minus HDL cholesterol. However, if TG ≥ 500 mg/dL, and particularly ≥1000 mg/dL, the focus of therapy shifts to acutely reduce TG in order to prevent pancreatitis. Thus, depending on the degree of HTG, therapeutic goals vary with variation in the emphasis of non-pharmacologic and pharmacologic therapeutic strategies (Table 5, Table 6). 

### 12.1. Non-Pharmacologic Management

Non-pharmacologic therapy is the only therapy required in patients with borderline-high TG levels (150–199 mg/dL) and here, achievement of the LDL cholesterol target is of primary importance. However, non-pharmacologic interventions [34] must be optimized, since HTG is often exacerbated by modifiable factors. Non-pharmacologic management includes: (1) strict glycemic control in patients with diabetes or impaired glucose metabolism; (2) treatment with levothyroxine in patients with hypothyroidism; (3) avoidance (if possible) of medications that increase TG (such as beta-blockers or thiazide diuretics); (4) limitation or abstinence of alcohol; (5) avoidance of simple carbohydrates; (6) low fat diet (<30% of total daily caloric intake) and when TG level >1000 mg/dL, a very low fat diet (<15% of total daily caloric intake); and (7) weight loss in patients who are overweight or obese [1]. 

Dietary intervention remains the mainstay of therapy. Patients with severe HTG with a monogenic basis should adhere to a stringent low fat diet (<15% of daily caloric intake from fat) [1,36]. Medium chain fatty acids can provide an alternate source of dietary fat given their direct absorption into the portal circulation with no reliance on chylomicron formation. Given this strict dietary regimen, supplementation with essential fatty acids (such as walnut oil or sunflower oil topically) [38] and fat soluble vitamins must be considered. In apo C-II deficiency, plasma transfusions have been shown to have transient benefit [39].

### 12.2. Pharmacologic Management

In addition to non-pharmacologic therapy, pharmacologic intervention using fibrates, statins, niacin, ezetimibe, or fish oil may be required if TG ≥ 200 mg/dL (Table 5). Furthermore, there are some special situations that call for particular treatment strategies (Table 6). Note that bile acid sequestrants should be avoided in patients with moderate to severe HTG due to their potential for further increasing TG levels.

### 12.3. Fibrates

In patients with TG ≥ 500 mg/dL, fibrates such as gemfibrozil, bezafibrate, and fenofibrate are the preferred pharmacologic therapy. Fibrates may also be used in patients with TG ≥ 200 mg/dL to help attain their non-HDL cholesterol target after they have met their LDL cholesterol target. 

Fibrates are weak agonists of peroxisome proliferator-activated receptor (PPAR)-α and lower TG by up to 50% by: (1) inhibiting hepatic synthesis and secretion of TG; and (2) stimulating degradation of TG-rich lipoproteins [40]. But while randomized clinical trials clearly demonstrate the TG-lowering efficacy of fibrates, they have shown inconsistent impact on reduction of CVD [41,42,43,44,45,46,47,48,49,50,51]. Addition of a fibrate to statin therapy seems to provide little additional benefit in CVD risk reduction.

For instance, the Action to Control Cardiovascular Risk in Diabetes (ACCORD) study [52] showed no additional cardiovascular benefit when adding fenofibrate to simvastatin therapy in patients with type 2 diabetes. These results were similar to those from the Fenofibrate Intervention and Event Lowering in Diabetes (FIELD) study [46] and the Bezafibrate Infarction Prevention (BIP) study [51]. A recent review indicated that fibrate therapy was associated with reduced risk for non-fatal myocardial infarction (odds ratio = 0.78; 95% confidence interval, 0.69–0.89) but had no effect on all-cause mortality [53]. In the ACCORD trial, there was a borderline significant trend towards cardiovascular benefit among the pre-specified sub-group analysis of patients with TG ≥ 204 mg/dL and HDL cholesterol ≤34 mg/dL when compared with other patients [52]. Other *post-hoc* and pre-specified sub-group analyses demonstrated cardiovascular benefit among patients with high TG levels, with or without low HDL cholesterol levels. Thus, fibrates in the high TG patient subgroup may warrant serious consideration.

### 12.4. Niacin

Niacin (nicotinic acid or vitamin B3, in high doses) is a therapeutic option for patients with TG ≥ 200 mg/dL who are unable to attain their non-HDL cholesterol goal, and also for patients with TG ≥ 500 mg/dL. The exact lipid-lowering mechanism of high-dose niacin remains unknown. Given crystalline niacin’s main side effect of flushing and vasodilation, extended-release preparations of niacin (ERN) are preferred for use compared to niacin or nicotinic acid. Doses of 500–2000 mg of ERN can lower TG by 5%–35% [54]. ERN also has additional dose-dependent beneficial effects on lipids, including lowering LDL cholesterol by 17%, lowering Lp(a) by 24%, and raising HDL cholesterol by 26% at the 2000 mg daily dose [54]. 

Niacin reduces cardiovascular events, as seen in the Coronary Drug Project, and also regresses plaques, as seen in the Familial Atherosclerosis Treatment Study (FATS), HDL Atherosclerosis Treatment Intervention Study (HATS), UC-San Francisco Specialized Center of Research (UCSF-SCOR) trial, Cholesterol Lowering Atherosclerosis Study (CLAS), and the randomized Arterial Biology for the Investigation of the Treatment Effects of Reducing cholesterol trials (ARBITER 2 and 3) [55,56,57,58,59,60]. However, the results of the recent Atherothrombosis Intervention in Metabolic Syndrome with Low HDL/High Triglycerides: Impact on Global Health Outcomes (AIM-HIGH) study indicate that addition of niacin to existing statin therapy in patients with high CVD risk does not further reduce CVD risk [61]. These results, together with the pending negative results of the Heart Protection Study 2: Treatment of High density lipoprotein to Reduce the Incidence of Vascular Events (HPS2-THRIVE) study have considerably diminished enthusiasm for niacin, at least when used in combination with a statin. However, niacin remains a suitable therapeutic option for some HTG patients, especially if a statin is not indicated or if the patient is intolerant of statin treatment.

### 12.5. Statins

Statins inhibit HMG-CoA reductase, the rate-limiting step in cholesterol biosynthesis, resulting in increased hepatic LDL receptor expression and enhanced cholesterol clearance from plasma. In addition, statins appear to have pleiotropic effects, including anti-inflammatory, anti-thrombotic, and anti-proliferative properties that may prevent plaque growth and rupture [62]. Unlike the other therapies discussed here, statins have proven cardiovascular morbidity and mortality benefit. 

In addition to their ability to lower LDL cholesterol, statins can also reduce TG levels by 10%–20% [63,64]. Statins are relatively ineffective in lowering TG among patients with severe HTG, but can help simultaneously achieve LDL cholesterol targets among patients with TG in the 200–499 mg/dL (2.3–5.6 mmol/L) range. 

Statins are efficacious in type 3 HLP since both TG and IDL are elevated. Combination therapy with a statin and fibrate can help normalize several components of the lipid profile, especially in patients with HLP type 2B and 3 [65,66]. In combination therapy with statins, fenofibrate is preferred to other fibrates, particular gemfibrozil, which has a higher rate of rhabdomyolysis when combined with a statin [67].

### 12.6. Ezetimibe

Ezetimibe is an intestinal cholesterol absorption inhibitor whose primary target is the NPC1L1 intestinal cholesterol transporter. Ezetimibe might be a useful adjunct therapy in patients with HTG. For instance, patients on a combination of fenofibrate 160 mg plus ezetimibe *vs.* fenofibrate alone had significantly greater TG reductions [68]. 

### 12.7. Omega-3 Fatty Acids

Omega-3 fatty acids (consisting of eicosapentaenoic acid (EPA) and docosahexaenoic acid (DHA)) probably lower TG by increasing glucose flux to glycogen, decreasing lipogenesis, and increasing mitochondrial β-oxidation [69]. According to NCEP ATP III guidelines, omega-3 fatty acids are recommended for patients with moderate to severe HTG [7]. At a dose of 4 grams/day, omega-3 fatty acids can lower TG by 20%–30% in patients with TG levels 200–500 mg/dL [70]. Plasma TG reductions of 50%–60% have been demonstrated among patients receiving 6 grams/day of omega-3 fatty acids [71]. The TG-lowering efficacy is related to baseline TG values [70]. Lower doses of omega-3 fatty acids (1 to 2 g/day) have also demonstrated significant reductions in cardiovascular mortality [72] and morbidity [73]. Thus, omega-3 fatty acids remain an important option in the management of HTG.

## 13. Conclusions

HTG is commonly encountered in clinical practice, and is a clinically relevant cause of acute pancreatitis. Recent genetic research has elucidated the polygenic nature of most cases of HTG, although the rare monogenic forms cause severe HTG and are informative biologically and biochemically. While epidemiologic evidence links HTG with CVD risk, randomized clinical trials using fibrates and niacin have not consistently shown reduction in CVD endpoints with treatment of HTG. Non-pharmacologic therapy is recommended to all patients with TG levels exceeding normal values while the decision to commence pharmacologic therapy depends on the degree of TG elevation. In addition to non-pharmacologic interventions, patients with TG > 1000 mg/dL are at risk of pancreatitis and warrant aggressive management using such therapeutic options as fibrates, niacin, and omega-3 fatty acids. Due to the uncertainty of clinical benefit, practice guidelines are not definitive regarding the management of patients with TG levels <500 mg/dL. Further work is required to define the benefits of treating patients with mild to moderate HTG.
